# Safety signals as instrumental reinforcers during free-operant avoidance

**DOI:** 10.1101/lm.034603.114

**Published:** 2014-09

**Authors:** Anushka B.P. Fernando, Gonzalo P. Urcelay, Adam C. Mar, Anthony Dickinson, Trevor W. Robbins

**Affiliations:** 1 Department of Psychology, University of Cambridge, Cambridge CB2 3EB, United Kingdom; 2 Behavioural and Clinical Neuroscience Institute, University of Cambridge, Cambridge CB2 3EB, United Kingdom

## Abstract

Safety signals provide “relief” through predicting the absence of an aversive event. At issue is whether these signals also act as instrumental reinforcers. Four experiments were conducted using a free-operant lever-press avoidance paradigm in which each press avoided shock and was followed by the presentation of a 5-sec auditory safety signal. When given a choice between two levers in Experiment 1, both avoiding shock, rats preferentially responded on the lever that produced the safety signal as feedback, even when footshock was omitted. Following avoidance training with a single lever in Experiment 2, removal of the signal led to a decrease in avoidance responses and an increase in responses during the safety period normally denoted by the signal. These behavioral changes demonstrate the dual conditioned reinforcing and fear inhibiting properties of the safety signal. The associative processes that support the reinforcing properties of a safety signal were tested using a novel revaluation procedure. Prior experience of systemic morphine during safety signal presentations resulted in an increased rate of avoidance responses to produce the safety signal during a drug-free extinction test, a finding not seen with *d*-amphetamine in Experiment 3. Morphine revaluation of the safety signal was repeated in Experiment 4 followed by a drug-free extinction test in which responses did not produce the signal for the first 10 min of the session. Instrumental avoidance in the absence of the signal was shown to be insensitive to prior signal revaluation, suggesting that the signal reinforces free-operant avoidance behavior through a habit-like mechanism.

The acquisition and persistence of avoidance behavior has been subject to much debate by learning theorists and clinicians as a symptom of phobias and anxiety disorders (Yehuda 2002; Abramowitz 2006; Lohr et al. 2007). Despite its relevance, free-operant avoidance has long been problematic for reinforcement theory as a successful avoidance response, by causing the omission of the negative reinforcer, is not followed by an observable event that can directly strengthen or reinforce the response. However, in a series of experiments conducted in the 1930s, Konorski and Miller (reported in Konorski 1948, 1967), observed that the performance of a spontaneous avoidance response led to inhibition of the Pavlovian defensive response that had been conditioned to a warning signal. Based on this finding, Konorski proposed a two-process theory of avoidance in which the conditioning of fear inhibiting, response-produced feedback stimuli provide a source of reinforcement for the avoidance response. The first, Pavlovian process consists of two components: (1) Excitatory aversive conditioning occurs to the context (and to any warning signal) due to presentations of an aversive negative reinforcer in that context and (2) as performance of the instrumental avoidance response prevents the aversive negative reinforcer, any feedback stimulus presented contingent with the avoidance response predicts the omission of the aversive reinforcer, thereby transforming it into a conditioned fear inhibitor and establishing it as a safety signal. The second, instrumental process is the reinforcement of the avoidance response by feedback or safety stimuli due to their fear inhibiting properties. Numerous theorists have subsequently advanced variants of this theory (e.g., Mowrer 1947, 1956; Dinsmoor 1954, 2001; Soltysik and Zielinski 1962; Bolles 1970; Denny 1971; Weisman and Litner 1972). Although evidence for the conditioned reinforcing properties of safety signals has been shown (Dinsmoor and Sears 1973; Morris 1975), what has not been previously investigated is the nature of the associative process mediating the reinforcing impact of safety signals.

It is now widely accepted that instrumental positive reinforcers operate through two processes (Dickinson 1985; de Wit and Dickinson 2009). The first establishes the instrumental response as a goal-directed action through the acquisition of a response-reinforcer or outcome (R–O) association. The canonical assay for the role of R–O associations in instrumental performance is the reinforcer or outcome revaluation procedure. Following instrumental training, the outcome is revalued in the absence of the opportunity to perform the instrumental response before performance of this response is tested in extinction. To the extent that instrumental responding is mediated by an R–O association, and therefore goal-directed, a change in the value of the outcome should produce a corresponding change in performance during the extinction test. In contrast, insensitivity to outcome revaluation in the extinction test indicates that performance is not mediated by a representation of the current value of the outcome or reinforcer, which is usually taken as evidence of control by the second, habitual process. Through this process, the outcome simply strengthens an association between stimuli present when the response is performed and the response-generation mechanism. As the outcome is not encoded within the stimulus–response (S–R) association, performance in the extinction test is impervious to changes in the value of the outcome following acquisition as long as the outcome itself is not presented during the test.

Our primary purpose was to investigate whether conditioned reinforcement of free-operant avoidance by a safety signal is goal-directed or habitual by analyzing the impact of revaluing the safety signal on avoidance responding in Experiments 3 and 4. As a prelude to these revaluation studies, however, we first established that a safety signal reinforces free-operant avoidance through the instrumental contingency between the signal and the avoidance response in Experiment 1. In the second experiment, we then showed that not only does the safety signal reinforce avoidance responding but, in accord with Konorski's (1948, 1967) theory, also functions as a conditioned inhibitor of avoidance.

## Results

### Experiment 1: Rats preferentially respond to produce the safety signal in a choice reversal test and in the absence of primary reinforcement by shock

Experiment 1 established that an instrumentally trained safety signal reinforces avoidance behavior using choice tests. We first trained the rats to press levers on two identical avoidance schedules before giving them a choice between the two levers, only one of which produced the safety signal. If the safety signal acts as a reinforcer, the rats should have preferred the lever that yielded this stimulus, despite the fact that both levers avoided the footshock reinforcer. In contrast, any noncontingent, general impact of the signal should have equally affected performance on both levers. The choice was then tested again in extinction in the absence of the primary reinforcer, a further test of the reinforcing properties of an instrumentally trained safety signal.

**Figure 1.**
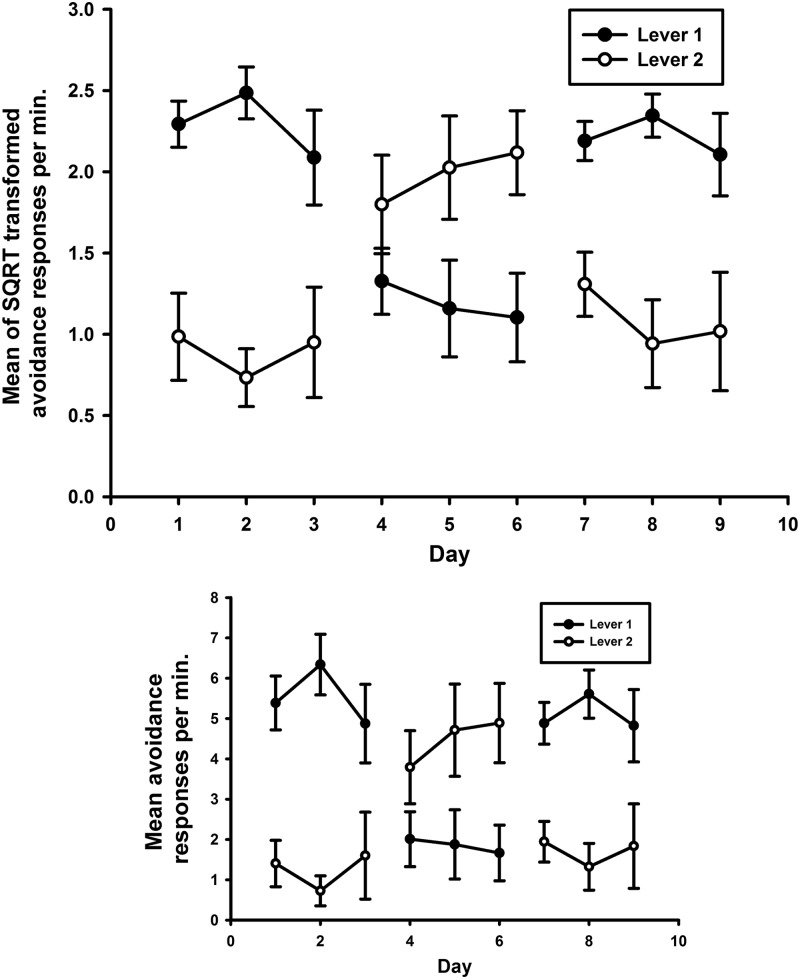
Rats preferentially responded to produce the safety signal in a two-lever choice test. (*Top*) Mean of the square-root transformed avoidance responses per minute on two levers with identical avoidance schedules; one of the levers produced the safety signal. Days 1–3 (Phase 1) the signal was presented contingent on responding on Lever 1, Days 4–6 (Phase 2) the signal was presented contingent on responding on Lever 2, Days 7–9 (Phase 3) the signal was presented contingent on responding on Lever 1. Each bar represents the mean of the SQRT transformed rate of avoidance responses per minute ± SEM. (*Bottom*) Mean untransformed avoidance responses per minute ± SEM.

Figure 1 shows that rats made more avoidance presses on the lever producing the safety signal (Lever 1 in Phases 1 and 3 and Lever 2 in Phase 2) than on the other lever in all three phases of the test. To evaluate this difference, the rates of avoidance responding on each lever were averaged across the three sessions of each phase before being evaluated by an analysis in which the lever variable contrasted performance of the lever producing the safety signal with performance on the other lever. A significant interaction was revealed between Phase and Lever (*F*_(2,12)_ = 18.8, *P* < 0.001) supporting the observation that lever preference switched with phase to produce the safety signal. No main effects were seen of Lever (*F*_(1,6)_ = 2.9, *P* = 0.1 NS) or of Phase (*F*_(2,12)_ = 1.5, *P* = 0.3 NS). Pairwise comparisons revealed a significant difference in responding between Levers 1 and 2 during Phases 1 and 3 (*P*’s < 0.05) but not during Phase 2 (*P* = 0.2 NS) which may have benefited from more training due to the change in lever on which the safety signal presentation was contingent on in this phase. The reinforcing effects of the safety signal observed in the reversal test under reinforcement were also present in the extinction test in that the means of the square root of avoidance responses per minute were 1.2 (SEM 0.2) for the safety signal lever, but only 0.6 (SEM 0.9) for the control lever, this preference being significant (*F*_(1,6)_ = 6.9, *P* < 0.04).

In summary, the pattern of avoidance responding during these two tests provides strong evidence that the safety signal functioned as a positive reinforcer. The preference for the lever producing the signal during the reinforced test shows the effect of the signal was mediated by the instrumental contingency. Moreover, the fact that the preference was also observed in the extinction test shows that the reinforcing properties of the signal were sustained in the absence of the primary aversive reinforcer just as in the case of conditioned reinforcers associated with appetitive reinforcement.

### Experiment 2: Reduced safety signal responding and increased avoidance responding reflect the inhibitory and reinforcing properties of the safety signal respectively on free-operant avoidance behavior

To the extent that the safety signal functions as a conditioned aversive inhibitor, we should expect its presentation to inhibit avoidance responding. To assess this prediction, following the first experiment we retrained the rats with the single avoidance response. On test sessions we omitted the safety signal following an avoidance response although each response continued to produce an unmarked safety period of the same duration as the signal (see Materials and Methods for further details). We compared the rate of responding during the safety signal in baseline sessions with the rate of responding during unmarked safety periods in the test session (rate of safety signal responses). During the same test sessions we also examined the rate of avoidance responding, responses that avoided shock and initiated the safety period, comparing baseline and test session rates. These test sessions were conducted following training with different durations of the shock-free avoidance period. To the extent that free-operant avoidance is motivated by aversive Pavlovian conditioning to the context, we should expect the rate of avoidance responding to decrease with longer avoidance periods as the reduction in the frequency of shock in the context should attenuate contextual conditioning. However, there are also reasons to anticipate that increasing the shock-free period after an avoidance response might facilitate responding by enhancing inhibitory conditioning to the safety signal and thereby its capacity to act as a conditioned reinforcer (Moscovitch and LoLordo 1968).

An analysis of the square-root transformed rate of responding during the last three sessions of each training phase with different shock-free avoidance intervals revealed no reliable effect of session (*F*’s_(2,12)_ < 1.4, *P* > 0.25) indicating that performance was stable at the end of training for each avoidance interval.

**Figure 2.**
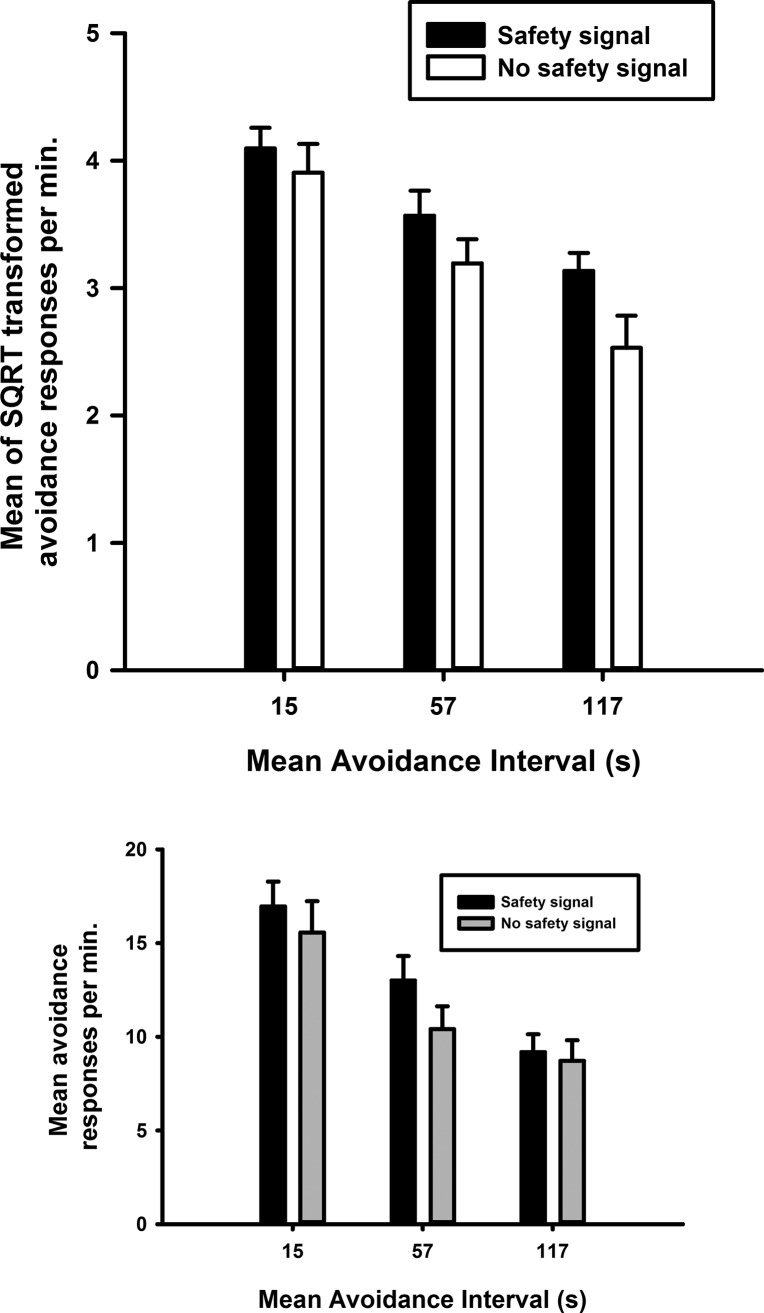
Avoidance responding was increased when reinforced by the safety signal. (*Top*) Mean of the square root transformed rate of avoidance responding, with the safety signal versus without the safety signal following training with three different avoidance intervals VI 15s, VI 57s, and VI 117s. Each bar represents the mean of the SQRT transformed rate of safety signal responses per minute ± SEM. (*Bottom*) Mean untransformed avoidance responses per minute ± SEM.

The rates of avoidance responding during the baseline (last training) session and the re-baseline session following the test session were averaged to yield a measure of responding with the safety signal which we compared with the rate during the test session when the safety signal was removed. Figure 2 illustrates that the rate of avoidance responding decreased as the mean avoidance interval increased (*F*_(2,12)_ = 86.6, *P* < 0.001) demonstrating that avoidance performance is sensitive to the frequency of shocks. This finding accords with the hypothesis that free-operant avoidance is motivated by Pavlovian aversive conditioning to the context which should increase with the density of shocks.

Importantly, Figure 2 also shows that the reinforcing effect of the safety signal observed in the first experiment, was replicated with a single response in that the rate of avoidance was higher in sessions with the safety signal at all intervals. There was a significant main effect of the presence of the signal (*F*_(1,6)_ = 10.5, *P* < 0.02) that did not interact significantly with the duration of the interval (*F*_(2,12)_ = 1.9, *P* = 0.2 NS).

**Figure 3.**
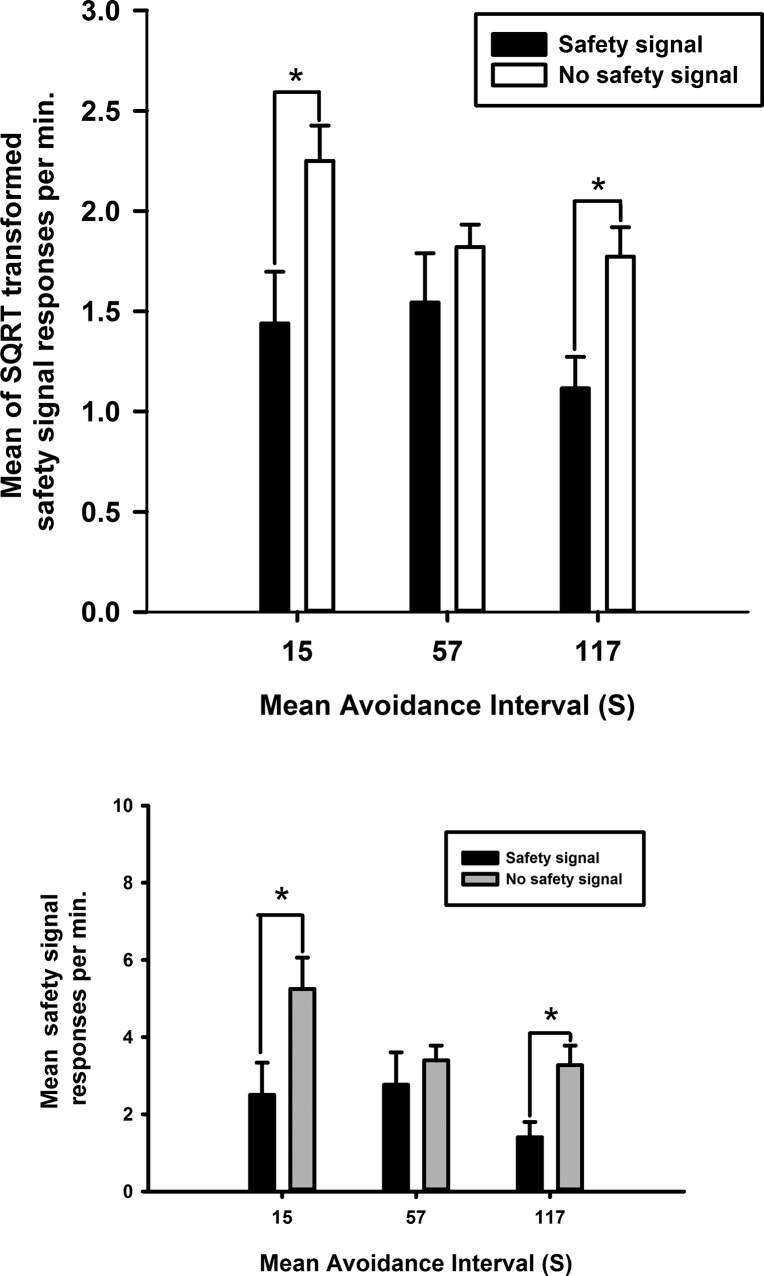
Loss of inhibition of responding in the absence of the safety signal. (*Top*) Mean of the square root transformed rate of safety signal responding, with the safety signal versus without the safety signal following training with three different avoidance intervals, VI 15s, VI 57s, and VI 117s. (*) *P* < 0.05 for responding with the safety signal versus without the safety signal. Each bar represents the mean of the SQRT transformed rate of avoidance responses per minute ± SEM. (*Bottom*) Mean untransformed safety signal responses per minute ± SEM.

Figure 3 illustrates that removal of the signal during the test sessions led to an increase in lever pressing during the safety period that followed each avoidance response (signal, *F*_(1,6)_ = 14.5, *P* < 0.01) suggesting that, when presented, the signal inhibited avoidance responding. However, the effect of removal of the signal differed between the avoidance intervals (avoidance interval × signal *F*_(2,12)_ = 6.8, *P* < 0.02) and was only reliable when the mean avoidance interval was short (15 sec) and long (117 sec) (pair-wise comparisons *P* < 0.01 for both intervals).

We did not anticipate that the inhibitory property of the safety signal would vary with the avoidance interval in this manner, with a greater inhibitory effect at both the shorter and longer intervals. In retrospect, however, this result is not theoretically anomalous. We have already noted that there are grounds for expecting inhibition to increase with the avoidance test period (Morris 1974). However, according to Rescorla and Wagner's (1972) account of inhibitory conditioning, the acquisition of conditioned inhibition depends on the level of excitatory conditioning when the inhibitor is presented without the reinforcer. We have already noted that the greater avoidance performance with shorter avoidance intervals suggests that contextual excitatory conditioning is greater with the shorter avoidance periods and therefore so should be inhibitory conditioning to the safety signal. Thus, it is reasonable to assume that inhibitory conditioning is influenced by two opposing processes in our procedure, being enhanced not only by shorter avoidance intervals due to the level of contextual excitatory conditioning, but also augmented by longer intervals due to the greater shock-free period predicted by the signal. These two influences appear to counteract each other at the intermediate avoidance interval, while allowing one or other of the processes to generate strong inhibition at the shorter and longer intervals.

### Experiment 3: Systemic morphine, but not *d*-amphetamine, revalued the safety signal, as evidenced by an increase in the rate of avoidance responding to produce the safety signal during a drug-free test

In this experiment we provide evidence that avoidance responding is sensitive to the current incentive value of the safety signal. The free-operant avoidance behavior trained in this study has two potential sources of reinforcement: (1) negative reinforcement arising from the negative contingency between the aversive footshock and the avoidance response and (2) positive reinforcement engendered by the positive contingency between the safety signal and avoidance response. Recently, we (Fernando et al. 2014) found that the negative reinforcer, footshock, can be revalued by presenting footshocks in the absence of the lever, when the rats are under the influence of morphine, *d*-amphetamine or central infusions of the µ-opioid agonist DAMGO (paired conditions). We therefore adopted an analogous procedure to revalue the safety signal, reasoning that due to the involvement of the opioid system in enhancing the hedonic impact of rewarding stimuli (Berridge 2003), this revaluation treatment should increase the value of the safety signal. Therefore, if the positive reinforcement provided by the safety signal depends upon its value, its ability to reinforce avoidance behavior should be enhanced following noncontingent exposure to the signal under morphine in the paired condition. Moreover, given that *d*-amphetamine potentiates responding for appetitive conditioned reinforcers (Taylor and Robbins 1984, 1986; Cador et al. 1991; Kelley and Delfs 1991), we also investigated whether presenting the signal under the influence of this drug would similarly augment its reinforcing capacity. The revaluation of the safety signal and the subsequent test of the impact of this revaluation on avoidance responding were conducted in the absence of the primary, footshock reinforcer specifically to assess the effects of the revaluation procedure on the reinforcing properties of the safety signal.

**Figure 4.**
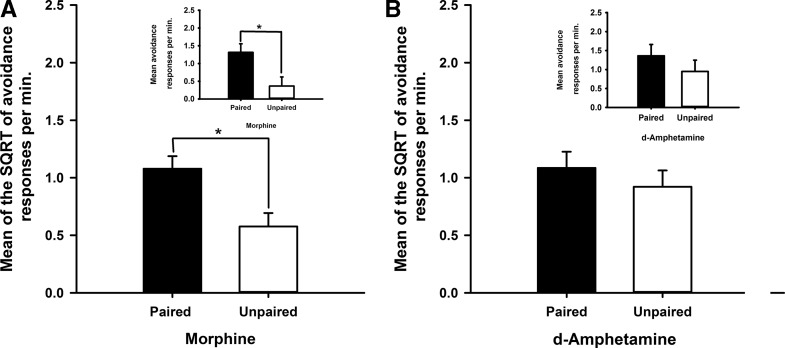
Systemic morphine revalued the safety signal. The effects of systemic (*A*) morphine and (*B*) *d*-amphetamine on the rate of avoidance responding during a drug free extinction test in rats that had received either prior pairings of the safety signal and drug (paired group) or unpaired presentations of the drug and safety signal across sessions (unpaired group) during the revaluation procedure. Each bar represents the mean of the SQRT transformed rate of avoidance responses per minute ± SEM. Graph *insets* depict the mean untransformed avoidance responses per minute ± SEM for each experiment.

Figure 4A illustrates that prior pairings of morphine with the safety signal during revaluation enhanced the rate of avoidance responding that produced the revalued safety signal during the subsequent drug-free test (paired group) with respect to a group that received unpaired injections of morphine and presentations of the safety signal during revaluation (*F*_(1,15)_ = 9.8, *P* < 0.01). This result suggests that prior pairings of morphine and the safety signal enhanced the value of the signal, thereby augmenting its ability to reinforce avoidance responding, even in the absence of shock. In contrast, as Figure 4B illustrates, pairing the safety signal with *d*-amphetamine had no detectable effect on its ability to reinforce avoidance responding (*F* < 1), suggesting a specific opioid-dependent mechanism for revaluation of the safety signal.

### Experiment 4: Prior revaluation of the safety signal with systemic morphine did not result in a change in avoidance responding during an extinction test in the absence of the revalued safety signal, suggesting habit-like mechanisms support avoidance behavior

In the final experiment, we exploited the finding in Experiment 3 that the opioid treatment revalued the safety signal, to investigate the associative process that supports the positive contingency between the avoidance response and safety signal. We did so by inserting an extinction test without the signal between the revaluation treatment and the reinforced test used in the previous experiment. As noted in the Introduction, an enhancement of avoidance responding in an extinction test without the safety signal indicates that responding is goal-directed with respect to the safety signal, and mediated by an R-O association. In contrast, if safety signals simply reinforce habitual responding; enhanced avoidance should only be observed when the response produced the signal in a reinforced test that was administered following the extinction test.

**Figure 5.**
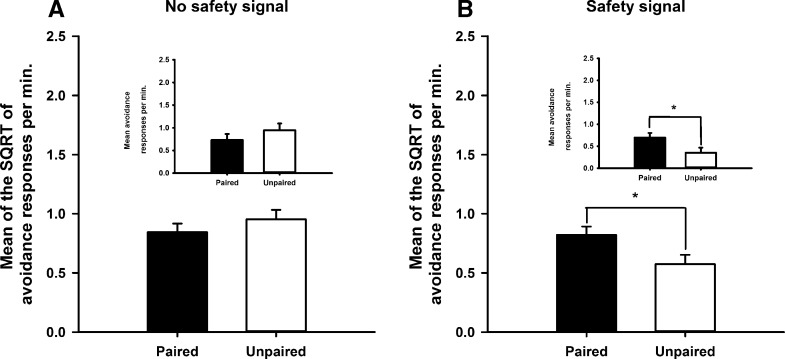
Habitual-like avoidance responding with respect to the positive contingency between the response and safety signal. (*A*) The effects of systemic morphine on the mean SQRT transformed avoidance responses per min during a drug-free extinction test. (*B*) The safety signal was then presented following lever press avoidance responses after the first 10 min of the extinction session. This test manipulation was conducted in two groups, the paired group that had received prior pairings of the safety signal and morphine or the unpaired groups where presentations of the safety signal and administration of morphine were across sessions. Each bar represents the mean of the SQRT transformed rate of avoidance responses per minute ± SEM. Graph *insets* depict the mean untransformed avoidance responses per minute ± SEM for each experiment.

Figure 5A illustrates that, in the absence of the safety signal, the paired and unpaired groups did not differ in their rates of avoidance responding during the drug-free extinction test. Figure 5B shows that once the safety signal was reintroduced during the reinforced test, responding was higher in the paired group than in the unpaired group. This description was confirmed by a significant interaction between revaluation treatment (paired versus unpaired) and the presentation of the safety signal (*F*_(1,9)_ = 8.2, *P* < 0.02) and pair-wise comparisons confirmed that there was a significant effect of revaluation (*P* < 0.05) in the reinforced test with the safety signal but not in the extinction test without the signal (*P* > 0.3). This pattern of results indicates that the safety signal acted by reinforcing habitual avoidance responding rather than being a goal of responding.

## Discussion

This study assessed the conditioned, functional properties of a safety signal and the associative processes that support its mediation of free-operant avoidance behavior in rats. The fear inhibiting and conditioned reinforcing properties of the safety signal were shown in both two-lever choice tests and single lever behavioral tests. The associative processes that support the ability of the safety signal to reinforce avoidance behavior were then tested using a novel revaluation paradigm where systemic morphine or *d*-amphetamine, paired with safety signal presentations, was predicted to enhance the incentive value of the safety signal. Although this revaluation treatment increased avoidance responding with a contingent safely signal, the absence of a comparable enhancement in the absence of the signal suggests that safety signals operate by reinforcing habitual avoidance responding. These results are discussed with reference to theories of the reinforcement of avoidance behavior.

### Safety signals reinforce avoidance behavior

Several lines of evidence support the conclusion that the safety signal reinforces free-operant avoidance behavior: (1) the preferential responding to produce the safety signal when the lever press-signal contingency was switched between levers as shown in Figure 1; (2) the continued preference for the safety signal lever in the absence of the shock in Experiment 1; and (3) the higher levels of avoidance responding maintained when the signal was presented during baseline sessions in Experiment 2, as shown in Figure 2. In this study, the presentation of the safety signal in the absence of shock following an instrumental avoidance response endowed the signal with fear inhibiting properties as manifested by a reduction in responding during the presentation of the safety signal (Fig. 3). This finding confirms previous demonstrations of aversive inhibition by safety signals following avoidance training (Rescorla 1969; Weisman and Litner 1969a,b; Morris 1975) and supports theories (Mowrer 1947, 1956; Konorski 1948, 1967) that argue their reinforcing properties arise from the Pavlovian inhibitory relationship between the safety signal and shock.

Despite evidence from previous studies and that presented in Experiments 1 and 2, demonstrations of the reinforcing properties of a safety signal have not always been successful (see Beck 1961; Lolordo 1969 for reviews). Fernando et al. (2013), failed to detect reinforcement of instrumental behavior by a safety signal when trained using a Pavlovian explicitly unpaired inhibition procedure. The safety signal did not support the acquisition of a new response, a stringent test of conditioned reinforcement, despite preferential responding seen in a separate group of animals for an equally trained appetitive stimulus that was previously paired with sucrose pellets. Demonstrations of the reinforcing properties of a safety signal have been achieved when the effects of a safety signal or conditioned fear inhibitor were assessed on an instrumental avoidance baseline (Moscovitch and Lolordo 1968; Rescorla 1969; Weisman and Litner 1969a,b, 1971; Dinsmoor and Sears 1973). The initial training of an avoidance response in these studies and the present study may have facilitated transfer of the inhibitory properties of the signal to the response so that it could act as a conditioned reinforcer. Moreover, conditioned reinforcement by an aversive conditioned inhibitor, such as a safety signal, may only be manifested in the aversive context engendered by an avoidance schedule, which was absent in the acquisition of a new response procedure.

The ability of a conditioned inhibitor of fear to reinforce instrumental avoidance behavior can be understood within the framework of appetitive-aversive interaction theory (Dickinson and Pearce 1977). This theory firstly assumes that there are two motivational systems, an appetitive and an aversive system. Dickinson and Dearing (1979) developed this theory by proposing that positive reinforcers have affective attributes that activate a central appetitive system, which mediates its reinforcing properties; similarly, negative aversive reinforcers activate a central aversive system. The second assumption is that these two systems reciprocally inhibit one another; the activation of one system thus results in the inhibition of the other. The theory therefore predicts that inhibition of the excited aversive system by the presentation of a safety signal results in rebound activation of the appetitive system through disinhibition. This activation of the appetitive system indirectly through disinhibition enables the safety signal to function as a positive reinforcer of the avoidance response, akin to the reinforcing properties of an appetitive stimulus. However, in contrast to an appetitive stimulus, the safety signal will only act as a positive reinforcer if the appetitive system is initially inhibited by the presence of an aversive context as reinforcement provided by a fear inhibitor is mediated by a rebound activation of the appetitive system. For this reason, fear provides the motivational prerequisite for the positive reinforcement engendered by a safety signal.

### Specific revaluation of the safety signal by morphine

Systemic injections of morphine before presentations of the safety signal (paired group), revalued the safety signal, resulting in a greater rate of avoidance responding to produce the safety signal during a drug-free test session. This selective increase in rate of avoidance responding suggests enhancement of the reinforcing properties of the safety signal on avoidance responding following revaluation with systemic morphine. Revaluation of the safety signal was not detected when pairing the safety signal with systemic *d*-amphetamine, suggesting the revaluation of a safety signal may require a specific opioid-dependent mechanism.

An analogous dissociation of the effects of morphine and *d*-amphetamine seen in this study can be found on feeding behavior. Both opioid and DA agonists have been shown to increase motivated behavior (Salamone et al. 1994; Wyvell and Berridge 2000; Zhang et al. 2003; Barbano and Cador 2006; Salamone and Correa 2012). The opioid system, however, has been uniquely identified in mediating the hedonic experience of palatable foods (Berridge 1996, 2000; Peciña and Berridge 2000). Opioid agonists have been shown to increase intake of highly palatable, sweet or fatty foods (Bakshi and Kelley 1993; Zhang and Kelley 1997), with opposing effects using opioid antagonists such as naltrexone (Yeomans and Gray 1997, 2002). In animals, palatability of food can be measured by observing changes in facial reactions which are believed to reflect core processes of positive hedonic impact and negative aversive impact which are conserved across species (Berridge 2000). “Liking” reactions, positive patterns of affective facial expressions to pleasant tastes (Grill and Norgren 1978; Berridge 2000) are unaffected by systemic administration of either DA antagonists (Treit and Berridge 1990; Peciña et al. 1997) or central infusions of *d*-amphetamine in the nucleus accumbens shell, a region shown to mediate hedonic taste reactivity (Wyvell and Berridge 2000). Morphine, however, has been shown to increase these reactions whether administered systemically, intraventrically or in the brain (Parker et al. 1992; Doyle et al. 1993; Peciña and Berridge 1995; Rideout and Parker 1996, Peciña et al. 2006). Thus, although morphine and *d*-amphetamine potentiate motivated behavior for appetitive rewards, only morphine enhances the hedonic value of the reward.

The effects of morphine and the µ-opioid agonist DAMGO have been described as producing a positive shift in affect across the hedonic spectrum as they enhance the pleasantness of sweet tastes and decrease the aversive properties of pain and bitter foods (Berridge 2003). Specific regions in the nucleus accumbens shell (NacS) and ventral pallidum known as “hedonic hotspots” have been identified as regions that mediate hedonic “liking” reactions which are predicted to reflect the pleasure experienced by an animal during consumption of sweet tastes (Peciña and Berridge 2000, 2005; Smith and Berridge 2007; Smith et al. 2011). Furthermore, infusions of DAMGO (a µ-opioid agonist) in the NacS were shown to enhance temporal firing of neurons in the ventral pallidum during the performance of these “liking reactions” and during the presentation of sucrose itself, suggesting a neural circuitry that mediates the hedonic experience of stimuli associated with reward (Smith et al. 2011). Similar regions have been shown to be activated with pain relief in humans (Zubieta et al. 2005; Leknes et al. 2011) consistent with the notion that relief is a hedonic experience (Gray 1987). A common hedonic circuitry may therefore mediate both pleasure of reward and relief from pain via activation of the opioid system.

### Safety signals reinforce habitual avoidance behavior

The drug-free test of Experiment 4 (Fig. 5A) showed that in the absence of the revalued safety signal, both the paired and unpaired groups responded at the same rate. Differences in the rates of avoidance behavior between revaluation groups would have been observed during the drug- free extinction test if avoidance responding had been mediated by a *representation* of the positive contingency between the avoidance response and safety signal and/or the current value of the safety signal. The failure to detect changes in the rates of avoidance responding was not due to a failure of the revaluation procedure. Once rats had experienced the revalued safety signal following the performance of the instrumental response during the reinforced test (Fig. 5B), a difference in the rate of avoidance responding was observed between the revaluation groups. The results of the reinforced test suggest that the revaluation of the safety signal with morphine was effective, replicating the results of Experiment 3. The results of the extinction test, however, lead to the conclusion that free-operant avoidance behavior is habitual with respect to the positive contingency between the response and safety signal. The safety signal, presented as a conditioned reinforcer of free-operant avoidance behavior, may have strengthened a direct association between the avoidance context and the response of lever pressing. Appetitive instrumental studies have shown that over-training of an instrumental behavior favors habitual control (Adams 1982; Dickinson et al. 1995; Tricomi et al. 2009). The habitual avoidance behavior shown in this study may have resulted from extensive training with the safety signal before revaluation; less avoidance training before the revaluation procedure could perhaps have produced goal-directed avoidance responding sensitive to changes in the value of the safety signal.

The effect of safety signal revaluation observed in Experiment 4 contrasts with our recent observation (Fernando et al. 2014) following revaluation of the primary negative reinforcer, the footshock, rather than the safety signal. After training on the same avoidance schedule as used in Experiment 4, we gave noncontingent exposure to the footshock under morphine before testing drug-free avoidance performance in an extinction test without shocks. In contrast to the insensitivity of extinction performance to safety signal revaluation, we found that morphine-based revaluation of the footshock reduced avoidance responding in the extinction test. A further apparent dissociation between control of avoidance by the primary and conditioned reinforcers was observed when revaluation occurred under *d*-amphetamine. Whereas exposure to the safety signal under this drug had no impact on subsequent avoidance responding in Experiment 3, revaluing the footshock with *d*-amphetamine reduced subsequent avoidance. This pattern of results suggests that the processes by which the primary and conditioned reinforcers impact on avoidance performance may well differ. As we have noted above, safety signals appear to establish habitual avoidance, whereas the primary reinforcer may well operate through a representation of at least some aspects of the avoidance contingency and in this sense be goal-directed.

In summary, the ability of a safety signal to reinforce free-operant avoidance behavior was shown in this study, as predicted by appetitive-aversive interaction theory. Its fear inhibiting properties were also revealed, supporting the two-process theory of avoidance behavior that predicts the reinforcing properties of a safety signal depend on it being a conditioned inhibitor of fear. Despite the sensitivity of free-operant avoidance behavior to the presence or absence of the safety signal and its modulation when presented with a revalued safety signal, instrumental avoidance was shown to be insensitive to revaluation of the safety signal when tested in the absence of this reinforcer. Similar to appetitive instrumental behavior that has been over-trained, the component of avoidance responding supported by the safety signal appeared to be mediated by S-R mechanisms when assessing the contingency between the avoidance response and safety signal. This habitual process, with respect to the safety signal and avoidance response, may well contribute to the persistent nature of avoidance behavior in extinction often observed by both theorists and clinicians. Considering the prevalence of safety signals in anxiety disorders (Rachman 1984; Woody and Rachman 1994; Carter et al. 1995), further studies of the conditioned properties of a safety signal and its neural mediation are needed. Cognitive behavioral therapy and therapeutics could then be developed specifically to prevent the accelerated development of habitual avoidance behavior in anxiety disorders, as a result of its reinforcement by safety signals.

## Materials and Methods

### Subjects

Subjects were experimentally naive, male, Lister-hooded rats, weighing ∼300 g at the start of the experiment and obtained from Charles River, UK. Rats were housed in groups of four per cage in a reverse light cycle room (12 h light:12 h dark; lights on at 0700) with water and food freely available throughout training and testing. Experiments 1 and 2 were conducted with the same eight rats; one rat was excluded from the study due to poor avoidance responding before the behavioral tests of Experiment 1. Experiment 3 was conducted with two groups sequentially, the morphine group consisted of 17 rats and the *d*-amphetamine group consisted of 16 rats. Experiment 4 was conducted in a group of 14 rats. Training and testing occurred during the dark phase and complied with the statutory requirements of the UK Animals (Scientific Procedures) Act 1986.

### Apparatus

Eight operant conditioning chambers (Med Associates, Vermont) each measuring 29.5×32.5×23.5 cm with a Plexiglas ceiling, front door and back panel and metal paneling on the sides of the chamber were used in Experiments 1 and 2. The same chambers plus six more of the exact same specification were used in Experiments 3 and 4. The floor of the chamber was covered with a metal grid with a metal tray beneath. Med Associates shocker generators (ENV-224AMWN, 115 V AC, 60 Hz) were connected to the metal grid and used to produce scrambled 0.5-sec, 0.5-mA foot shocks. Each chamber was placed within a sound- and light-attenuating box and interfaced to a computer through Whisker control software (Cardinal and Aitken 2010). The safety signal was a 2900-Hz tone produced by a Med Associate tone generator (ENV-223AM) for half of the rats and a white noise by a Med Associate white noise generator (ENV-2255M) for the remaining rats. Both these generators were attached to the same wall of the chamber, which was opposite to the wall from which the levers extended. Stimuli were set to 8 dB above background level. Levers could be extended either side of a central food magazine on the opposite side wall, but no pellets were ever delivered.

### Behavioral procedures

#### Pretraining

Rats were first habituated to the chamber and the levers for 4 d. For the first 2 d either the left or the right lever was randomly chosen at the start of the session. This chosen lever was then extended at the start of the session and any responses resulted in its retraction followed by its immediate extension back into the chamber. For the last 2 d the opposite lever was extended and the number of responses was limited so that the number of retractions and extensions of the two levers was equated; houselights remained on until the end of the 1 h session. Each daily session lasted 1 h.

#### Training

The start of the session was marked with the illumination of the houselight and the extension of a single lever, which was randomly chosen as either the right or the left lever at the start of the session. This lever remained permanently extended for the entire session. The session began with an unsignaled avoidance period of 60 sec, and in the absence of a lever press was followed by intermittent foot shocks. During this shock period the shock-shock interval was 10 sec. After five presentations of shock, the shock period terminated automatically and was immediately followed by the next avoidance period. The maximum number of shocks in the session was limited to 30 at which point the session ended. Any lever press during the avoidance or shock periods immediately terminated these periods with a 60-sec auditory safety signal, which was then followed by the next avoidance period. Across sessions the levers were randomly switched to either right or left on a daily basis, so they were equated in experience for all stages of training. Lever presses during the signal had no consequence and did not contribute to the assessment of avoidance responding. The durations of the avoidance period and the safety signal were gradually reduced across training to the final values of 15 and 5 sec, respectively, for Experiments 1 and 2. Following the two-lever tests conducted in Experiment 1, rats were trained on a single lever across successive phases with variable avoidance periods with the following averages (range): 56.5 (3–110 sec), 116.5 (3–230 sec), and 15 sec (3–27 sec) for Experiment 2. Training continued on the given avoidance schedule until responding was stable for three days and was then immediately followed by a test session in each phase.

An optimized training procedure was used in Experiments 3 and 4 in two separate cohorts in which durations of the avoidance period and safety signal were gradually increased and decreased, respectively, across stages of training. As for the rats of Experiments 1 and 2, the start of the session was marked with the illumination of the houselight and the extension of a single lever, which was randomly chosen as either the right or the left lever at the start of the session. This lever remained permanently extended for the entire session. The training procedure differed to that previously reported as the session began with an unsignaled avoidance period that varied between 120 and 140 sec, and in the absence of a lever press response, was followed by intermittent foot shocks (0.2 mA). During this shock period the mean shock-shock interval was 3 sec (range 1–5 sec). After three presentations of shocks, the shock period terminated automatically and was immediately followed by the next avoidance period. The maximum number of shocks that could be presented in the session was limited to 30 at which point the session ended. Any lever-press during the avoidance or shock periods immediately terminated these periods with a 120 sec auditory safety signal, which was then followed by the next avoidance period. Across sessions the levers were randomly assigned to either right or left on a daily basis, so they were equated in experience for all stages of training. Lever presses during the signal had no consequence and did not contribute to the assessment of avoidance responding. The durations of the avoidance period and the safety signal were gradually reduced and the shock intensity increased in 0.1 mA increments across training to the final mean values of 120 sec (range 10–230 sec) and 5 sec and 0.5 mA, respectively. The final training parameters were chosen based on the results of Experiment 2 where both the inhibitory and reinforcing properties of the safety signal were observed with these parameters. Subjects in all experiments were trained for ∼1 mo until they reached the final stage of training.

**Figure 6.**
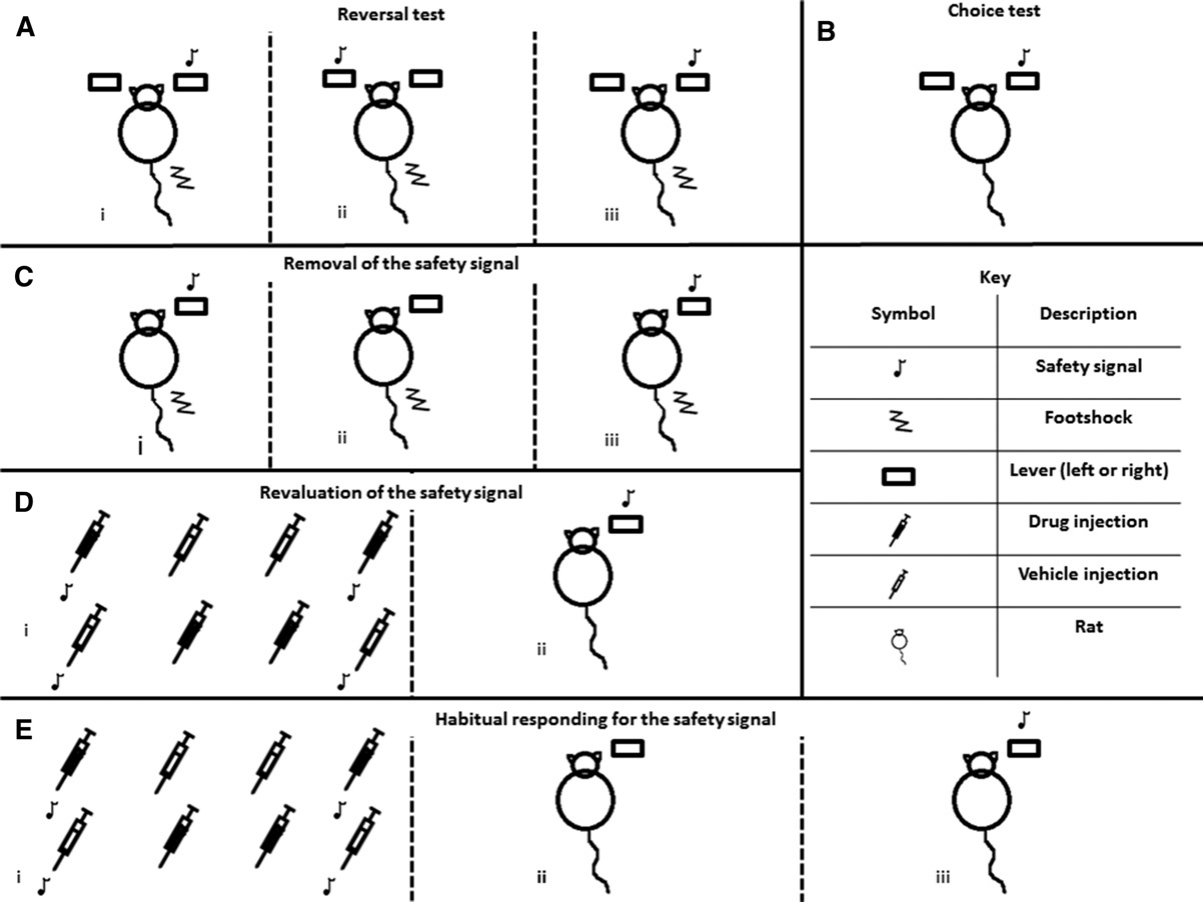
Schematics of experimental procedures. (*A*) Experiment 1: reversal test, two levers are presented which both avoid shock but only one produces the safety signal (i) for the first three days (Phase 1) this is presented following responding on the same lever (either right or left counterbalanced across subjects), (ii) for the next three days the safety signal is presented following responding on the alternate lever to that of the first three days (Phase 2), (iii) the safety signal is presented following responding on the same lever as used in the first three days for three more days (Phase 3). (*B*) Two-lever choice test where one lever produces the safety signal but no shocks are presented in the session. (*C*) Experiment 2: removal of the safety signal on avoidance responding, (i) baseline session, responses avoid shock and produce the safety signal, (ii) responses avoid shock but do not produce the safety signal, (iii) responses avoid shock and produce the safety signal. (*D*) Experiment 3: revaluation of the safety signal, (i) revaluation procedure; (*top* line) the paired group; (*bottom* line) the unpaired group, (ii) drug-free test session where responses on a single lever produce the revalued safety signal, no shocks are presented during the revaluation and extinction test sessions. (*E*) Experiment 4: habitual avoidance behavior test, (i) revaluation procedure; (*top* line) the paired group; (*bottom* line) the unpaired group, (ii) drug-free test session where responses on a single lever do not produce the revalued safety signal, and (iii) reinforced test where responses on a lever produce the revalued safety signal; no shocks are presented during revaluation or test sessions.

The behavioral tests used in each experiment are illustrated in Figure 6.

### Experiment 1: reversal test

During each test session both levers were inserted into the chambers and a press on either lever functioned as an effective avoidance response under the schedule established at the end of the training phase (avoidance period of 15 sec followed by five shocks with a shock–shock interval of 10 sec). However, only presses on one of the levers produced the safety signal (signal responses) and the side of the initial signal responses was counterbalanced across rats. A press on the nonsignal lever resulted in the same 5-sec period of safety but did not produce the auditory safety signal. Therefore, an avoidance response on either the signal or the nonsignal lever during the avoidance or shock periods instituted a 5-sec period during which presses on the levers were neither effective nor contributed to the assessment of avoidance performance. The next avoidance period started immediately following this 5-sec period. The lever associated with the signal remained the same for three sessions before being switched to the alternate lever for the next three sessions, Finally, the presses on the original lever produced the signal for the last three sessions, thereby generating an ABA design across the three phases of the test.

### Experiment 1: test session

The procedure remained the same as during the reinforced test except the lever assigned for signal response varied randomly across sessions to prevent any lever biased responding. This training was continued until there was significant preference for the lever associated with the signal (regardless of whether this was the left or right lever) for three of these baseline sessions. A single 1-h test session was then conducted in which there were no shocks and each response on the signal lever produced the 5-sec safety signal. This lever was again randomly chosen before the start of the session.

Performance was then tested under varying doses of systemic *d*-amphetamine, but these are not reported because the drug did not reliably affect responding.

### Experiment 2: removal of the safety signal on avoidance responding

Testing started with a baseline session with the same procedure as during training. This schedule also remained in force on the next day during the test session except that the safety signal was omitted following an avoidance response, despite the response avoiding shock and producing a 5-sec unsignaled period of safety. Two rates of responding were analyzed during the test sessions: the rate of avoidance responding; responses that avoided shock and produced the safety period divided by the total avoidance time; and the rate of safety signal responding, responses that occurred during the safety period which are nonconsequential divided by the total safety time. These tests on a single lever were conducted following training with different variable shock-free avoidance periods. Test sessions were conducted once a stable baseline level of avoidance behavior was observed with the current shock-free avoidance period schedule. Following the test session subjects were returned to the same baseline condition before the test session for 1 d. Rates of responding on the baseline day before and post the test session were included in the analyses.

### Experiments 3 and 4: revaluation procedure

The revaluation procedure lasted 4 d (one session per day) and differed between the paired and unpaired groups. Rats in the paired group received two sessions where the analgesic drug was administered before a session in which 15 presentations of the trained safety signal were experienced in the absence of the lever and shock. The safety signal was presented for 5 sec after a mean variable interval of 90 sec (range of 60–120 sec). During the other two sessions, rats received vehicle injections before sessions where nothing occurred in the chamber for the 30 min. In the unpaired group, the drug was administered before sessions where nothing occurred in the chamber and vehicle was administered before sessions with safety signals. The only difference between these two revaluation groups was therefore the contingency between the injections of drug and the presentations of the safety signal.

### Experiments 3 and 4: drug administration

All rats received 4 d of intra-peritoneal injections, 2 d with the drug (morphine 10 mg/kg or *d*-amphetamine 1.5 mg/kg, calculated as free base) and 2 d with vehicle (0.9% filtered saline). Doses were chosen for their analgesic effects in the absence of motor depressant effects (morphine: Babbini and Davis 1972; Babbini et al. 1979; Kuribara et al. 1985; *d*-amphetamine: Abbott and Guy 1995; Fernando et al. 2013).

### Experiment 3: test session

Following the 4-d revaluation procedure a single drug-free test session was conducted which was the same as baseline training sessions except in the absence of the footshock. Responses on a single lever therefore produced the auditory stimulus (white noise or tone) for 5 sec during the 30-min test session.

### Experiment 4: extinction test + reinforced test

The drug-free test session differed to that of Experiment 3 as for the first 10 min of the session the safety signal was not presented following responses on the lever (extinction test). After these first 10 min, the safety signal was then presented for the rest of the session following avoidance responses on the lever (reinforced test). No shocks were presented during this test session which lasted for 30 min.

### Data analysis

Responses on the lever made to avoid or escape shock (therefore outside the 5-sec safety period) were taken as a measure of the rate of avoidance responding. This division of responding has been used in previous studies to assess the reinforcing properties of a safety signal on avoidance behavior (Rescorla and Lolordo 1965; Dinsmoor and Sears 1973). Responses made during the 5 sec following a lever press response were taken as the rate of safety signal responses; these responses were nonconsequential and taken as a measure of the inhibitory properties of the signal. Both forms of responding were square root transformed for statistical analysis.

### Statistical analysis

Within-subject analyses of variance (ANOVAs) were conducted with a rejection criterion of *P* < 0.05 and, where necessary, the Huynh–Feldt adjustment was applied if sphericity was violated. This was conducted for all experiments in this study. In the presence of interactions, pairwise comparisons were also conducted and adjusted using the Sidak correction.

## Competing interest statement

T.W.R. is a consultant for Cambridge Cognition, Eli Lilly, GSK, Merck, Sharpe and Dohme, Lundbeck, Teva, and Shire Pharmaceuticals. He is or has been in receipt of research grants from Lundbeck, Eli Lilly, and GSK and is an editor for Springer-Verlag. All other authors report no conflicts of interest.
